# Unilateral ophthalmoplegia in anti-GQ1b antibody syndrome: case report and systematic literature review

**DOI:** 10.3389/fimmu.2025.1669821

**Published:** 2025-10-10

**Authors:** Juyuan Pan, Ningyu Zheng, Dan Yu, Huihua Jiang, Yuanlin Zhou

**Affiliations:** Department of Neurology, Taizhou Hospital of Zhejiang Province Affiliated to Wenzhou Medical University, Linhai, Zhejiang, China

**Keywords:** unilateral ophthalmoplegia, anti-GQ1b antibody syndrome, case report, systematic literature review, Miller Fisher syndrome

## Abstract

**Introduction:**

Anti-GQ1b antibody syndrome encompasses immune-mediated neuropathies targeting ganglioside GQ1b, classically presenting as Miller Fisher syndrome (MFS) with the triad of ophthalmoplegia, ataxia, and areflexia. While bilateral ophthalmoplegia is typical, unilateral presentations represent a recognized variant phenotype.

**Methods:**

We report a case of a 17-year-old male with unilateral complete oculomotor nerve palsy, confirmed by positive serum anti-GQ1b IgG antibodies. We also conducted a literature review identifying 17 additional cases of anti-GQ1b associated unilateral ophthalmoplegia, summarizing clinical features, investigations, management, and outcomes for all 18 patients.

**Results:**

Among the 18 patients, a male predominance was observed (13 males, 5 females), with a median age of 31 years (range 10-68). Most patients (17/18, 94.4%) reported a preceding illness. Unilateral external ophthalmoplegia was universal, most commonly affecting adduction (55.6%) and vertical gaze (55.6%). Internal ophthalmoplegia (IO) occurred in 6 cases (33.3%), including 4 unilaterally. Cerebrospinal fluid (CSF) protein was mildly elevated (23–97 mg/dL) in 7 of 18 cases. Treatments varied and prognosis was uniformly favorable, with most patients recovering within 3 months.

**Conclusion:**

Unilateral ophthalmoplegia, particularly when complicated by ipsilateral internal ophthalmoplegia, constitutes a distinct regional variant of anti-GQ1b antibody syndrome. Early serological testing for anti-GQ1b antibodies is key to diagnosis, especially in patients with antecedent infection.

## Introduction

Anti-GQ1b antibody syndrome represents an expanding spectrum of immune-mediated neuropathies unified by seropositivity for anti-GQ1b IgG antibodies. Initially described in classic Miller Fisher syndrome (MFS) characterized by the triad of ophthalmoplegia, ataxia, and areflexia, this syndrome now encompasses diverse phenotypes including Bickerstaff brainstem encephalitis, Guillain-Barré syndrome with ophthalmoplegia, and acute isolated ophthalmoplegia without ataxia (1). A key pathophysiological feature is the selective targeting of ganglioside GQ1b, highly expressed​​ in the paranodal regions of the oculomotor, trochlear, and abducens nerves within cranial nerves (2). Notably, ophthalmoplegia may manifest asymmetrically or even monocularly in 27% of patients, with patterns ranging from isolated nerve palsies to complete external/internal ophthalmoplegia (3). ​​Adding to this clinical heterogeneity, we report a distinctive case of unilateral complete oculomotor nerve palsy result from anti-GQ1b antibody syndrome.

## Case report

A 17-year-old male presented with a 4-day history of progressive right ocular pain, photophobia, diplopia, and right eyelid ptosis. Two weeks prior to admission, the patient experienced an upper respiratory tract infection characterized by fever, cough, and productive sputum, which resolved with self-administered medication. Initial ophthalmologic evaluation demonstrated corrected visual acuity of 1.0/1.0 in both eyes with intact color vision. Intraocular pressures were 13 mmHg (right) and 13.5 mmHg (left). A fixed dilated right pupil measuring 5 mm with absent light reflex was observed, accompanied by right eyelid ptosis.​​ The left pupil was normal in size (3 mm) and reactivity. Extraocular motility examination showed limitation of adduction (right medial rectus muscle), elevation (right superior rectus muscle), and depression (right inferior rectus muscle).

**Figure 1:**
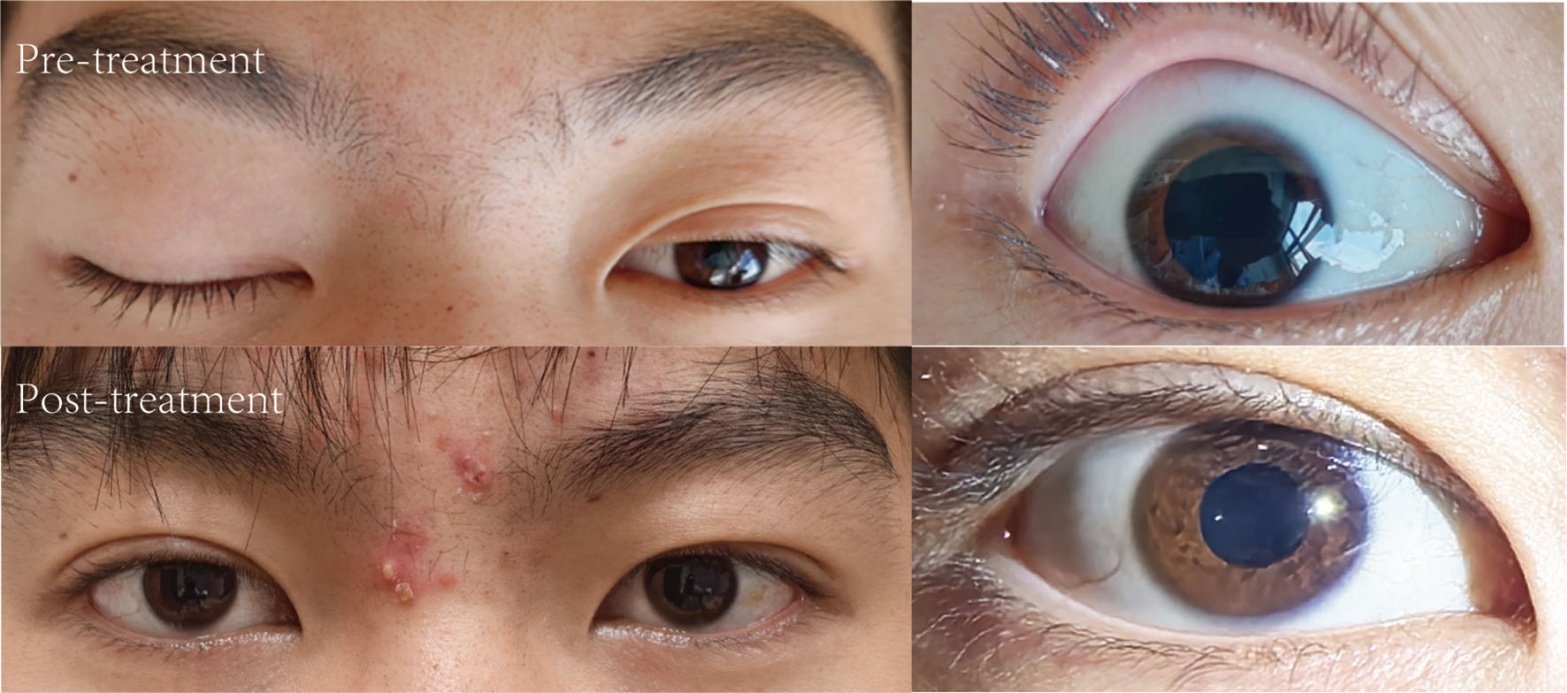
Pre- and post-treatment comparison of right oculomotor nerve palsy with ptosis and Pupillary Abnormality.

The patient was subsequently admitted to the neurology department for further evaluation and management. Repeat neurologic assessment confirmed persistent right oculomotor nerve palsy. Systemic neurological examination, including assessment for ataxia, deep tendon reflexes, and other cranial nerves, was otherwise normal. Comprehensive laboratory investigations, including complete blood count, C-reactive protein, and serology for HIV and syphilis, yielded results within normal limits. Lumbar puncture revealed a cerebrospinal fluid (CSF) white blood cell (WBC) count of 4 *10^6/L, protein level of 36 mg/dl on day 7. Neuroimaging with computed tomography angiography (CTA) of the head showed no evidence of aneurysm or vascular abnormality. Contrast-enhanced magnetic resonance imaging (MRI) of the brain was unremarkable. Serum testing was positive for anti-GQ1b IgG antibodies. Initial management included neuroprotective therapy (Mecobalamin capsules and Vitamin B1 tablets) and intravenous methylprednisolone sodium succinate (80 mg once daily). While ocular pain subsided, diplopia and ptosis persisted. Subsequent serum re-testing showed positive anti-GQ1b IgG antibodies. The patient then received a 5-day course of intravenous immunoglobulin (IVIg) at 400 mg/kg per day. Oral corticosteroid therapy was subsequently initiated and gradually tapered. Two weeks after discharge, digital subtraction angiography(DSA) was performed at another institution to exclude vascular pathology such as aneurysm, revealing no abnormalities.​ At the 3-month follow-up, the patient exhibited progressive improvement in ocular motility and pupillary function without recurrence or development of classic MFS features (**Figure 1**).

## Results: literature review and our case

To obtain current and relevant data, including detailed clinical information for each case, we searched the PubMed database from January 2000 through June 2025 for full-text case reports and case series in English. The **Table 1** summarizes the clinical characteristics, lab findings, management, and outcomes of 18 patients with unilateral ophthalmoplegia associated with anti-GQ1b IgG antibodies, including the present case. Patients ranged in age from 10 to 68 years (median age 31 years), with a male predominance (13 males, 5 females). Most patients (17/18) reported a preceding illness, including upper respiratory tract infection, diarrhea, asymptomatic chlamydia pneumoniae infection, campylobacter infection, parvovirus B19 and COVID-19 vaccination. Neuro-ophthalmologic examination revealed ptosis in 8 cases (44.4%), and all patients exhibited unilateral external ophthalmoplegia (EO), predominantly involving limitations in adduction (10/18, 55.6%), vertical (10/18, 55.6%) and abduction (9/18, 50%) gaze. Internal ophthalmoplegia (IO) was present in 6 cases (31.6%), with 4 cases exhibiting unilateral involvement​. Deep tendon reflexes were reported as absent in 3 patients, decreased in 5, and normal or not specified in others. Ataxia was present in 3 patients. CSF analysis showed normal or mildly elevated white blood cell counts (WBC) (range: 0-12.8 x 10^6/L) and normal or mildly elevated protein levels (range: 23–97 mg/dL). MRI findings revealed nerve enhancement in two patients, with one showing enhancement in the abducens nerve and the other in the oculomotor nerve. ​Electrophysiological data were available for 7 of the 18 reviewed patients. Nerve conduction studies were normal in 6 cases, while one patient exhibited reduced F-wave frequency (18%, normal >40%) in the left ulnar nerve (4). Treatment approaches varied. Conservative management or no specific immunomodulatory treatment was used in 4 cases. Immunotherapy included IVIg in 4 patients, corticosteroids in 6 patients, and sequential therapy (corticosteroids followed by IVIg) in 2 patients. Prognosis was generally favorable, with most patients achieving recovery. Recovery times varied, reported from as early as 28 days to within 3 months. One case had partial recovery within 1 month, one case reported follow-up loss, and outcomes were not available for several cases from the Yuki et al. series (5).

**Table 1:** Clinical features of patients with unilateral ophthalmoplegia due to anti-GQ1b antibody syndrome.

No	Age/ Sex	Preceding illness	Neuro-ophthalmologic examination			Deep tendon reflex	Ataxia	CSF findings cell count (ml)/protein (mg/dl)	MR finding	Treatment	Prognosis
			Ptosis	EO	IO						
Susuki (15)	35/M	Cough and sore throat	Left	Left horizontal and vertical	–	Absent	+	normal/56	normal	None	Recovery within 3 months
Vanden (16)	12/M	Asymptomatic Chlamydia pneumoniae infection	No	Left abduction	–	–	–	normal/97	normal	None	Partial recovery within 1 month
Yuki (5)	26/F	Sore throat	No	Left abduction	–	–	–	0/24	Enhanced in abducens nerve	Na	Na
	32/M	No	No	Left abduction	–	Decreased	–	1/41	normal	Na	Na
	26/F	URI	No	Left abduction and adduction	–	Decreased	–	2/34	normal	Na	Na
	35/M	URI	No	Left abduction and down	–	–	–	3/51	normal	Na	Na
	18/F	Fever and headache	No	Right horizontal and vertical	–	Decreased	–	0/30	normal	Na	Na
(17)	47/M​	Fever and cough	Left	Left adduction and vertical	Left	–	–	normal/80	normal	Oral prednisolone (not effective) and IVIG on day 21)	Recovery within 28 days
Smith (18)	32/M	Mild coryzal illness	Left	Left horizontal and vertical	–	Absent	+	​​normal/normal​	normal	Conservative management	Recovery within five weeks
Lee (3)	30/M	URI	Right	Right adduction and vertical	–	Na	–	0/57	normal	Na	Recovery within 3 months
	27/M	Diarrhea	No	Right vertical	Bilateral	Na	–	0/23	normal	Na	Lost to follow-up
	53/M	URI	Left	Left adduction and vertical	–	Na	–	0/34	normal	Na	Recovery within 3 mo
Kinno (4)	27/M​	Cough and sore throat (Haemophilus parainfluenzae)	No	Right abduction	–	Absent	–	normal/62​	normal	​​IVIG	Recovery within 52​ days
Ueno (19)	68/M	Campylobacter infection	Right	Adduction and vertical	-​	Decreased	–	2/40 (normal)​	normal	Conservative treatment	Recovery within 87 days
Canavese (20)	10/F	Flu-like syndrome(parvovirus B19)	Left	Left inferior oblique muscle	Left	–	–	normal	normal	Oral prednisone	Recovery within 1.5 months
Kubota (21)	65/M	COVID-19 vaccination	No	Right adduction and vertical	Right	–	–	2/52	Enhance in oculomotor nerve	IVIG	Mild improvement by day 36; ptosis resolved by day 52; complete recovery by day 71
Canavese (22)	53/F	Cough and fever	No	Right adduction and infraduction	Bilateral	–	–	12.8/33.3	normal	IV dexamethasone (15mg)	Recovery within 42 days
Our case	17/M	URI	Right	Adduction and verticale	Right	–	–	4/36	normal	IV methylprednisolone and IVIG	Recovery within 3 months

EO, External ophthalmoplegia; IO, Internal ophthalmoplegia; L, Left; R, Right; URI, Upper respiratory tract infection; IV, Intravenous; IVIG, Intravenous immunoglobulin.

## Discussion

We reported a case of a 17-year-old male who developed progressive right ocular pain, photophobia, diplopia, and ptosis four days after recovering from an upper respiratory infection. Neurological examination revealed isolated right oculomotor nerve palsy, manifesting as ptosis, impaired adduction, elevation, and depression; and a fixed, dilated right pupil. Comprehensive diagnostic work-up, including normal neuroimaging excluded compressive or vascular lesions. The diagnosis of anti-GQ1b IgG antibody syndrome was confirmed by positive serum anti-GQ1b IgG antibody testing. Initial management with corticosteroids provided only partial relief. The patient was then treated with IVIg followed by a tapering course of oral corticosteroids, which led to progressive improvement. At the 3-month follow-up, ocular motility and pupillary function had recovered. This case exemplifies the rare presentation of unilateral complete oculomotor nerve palsy as a regional variant within the anti-GQ1b antibody syndrome spectrum.

Our case of unilateral oculomotor palsy with internal ophthalmoplegia represents a distinct variant within the expanding spectrum of anti-GQ1b antibody syndrome. Historically, Miller Fisher syndrome (MFS), characterized by the classic triad of ophthalmoplegia, ataxia, and areflexia, and Bickerstaff brainstem encephalitis (BBE), defined by ophthalmoplegia, ataxia, and impaired consciousness or hyperreflexia, were considered distinct entities. However, the discovery of serum IgG anti-GQ1b antibodies as a highly sensitive and specific serological marker common to both conditions, along with shared antecedent infections and overlapping clinical features, has led to the recognition of a continuous spectrum now termed “anti-GQ1b antibody syndrome”. Within this spectrum, classic MFS primarily manifests with peripheral nervous system involvement (oculomotor nerve dysfunction, sensory ataxia due to muscle spindle afferent involvement, and areflexia), while BBE represents the end with significant central nervous system involvement, evidenced by altered consciousness, hyperreflexia, or Babinski signs, potentially due to disruption of the blood-brain barrier allowing antibody access to brainstem structures like the reticular formation. Our case of unilateral ophthalmoplegia, especially with ipsilateral internal ophthalmoplegia, aligns with recognized variant phenotypes within this syndrome. Beyond MFS and BBE, the anti-GQ1b antibody spectrum encompasses forme fruste presentations such as acute isolated ophthalmoplegia without ataxia/areflexia, acute ataxic neuropathy without ophthalmoplegia, pharyngeal-cervical-brachial weakness, and overlaps with Guillain-Barré syndrome.

The pathogenesis of Anti-GQ1b IgG antibody syndrome is intricately linked to antecedent infections, with molecular mimicry serving as a central mechanism. As demonstrated in our case, the patient developed an upper respiratory tract infection (URI) preceding ophthalmoplegia. ​​This observation was consistent with broader literature data, where most patients with anti-GQ1b-associated unilateral ophthalmoplegia reported antecedent illness.​ Campylobacter jejuni was the most well-established pathogen associated with MFS, implicated in 21% of cases (6). Its lipo-oligosaccharide mimicked GQ1b ganglioside structures, inducing cross-reactive antibodies that attack neural tissues. Notably, strains harboring the cst-II (Asn51) gene polymorphism frequently expressed GQ1b-like epitopes and were overrepresented in MFS (7). Beyond Campylobacter jejuni, other pathogens such as Haemophilus influenzae exhibited similar molecular mimicry. Despite these associations, our case and literature revealed that 67% of MFS patients have no identifiable antecedent pathogen. This may reflect limitations in pathogen detection or the involvement of host-specific factors. Genetic susceptibility, such as HLA-DR2 alleles, was linked to recurrent MFS (1). Furthermore, Taboada et al. highlighted that neuropathogenic and enteritis-associated C. jejuni strains share high genomic similarity, suggesting that host immune responses—rather than pathogen virulence alone—determined clinical outcomes (8). For example, impaired blood-nerve barrier integrity may facilitate antibody access to neural targets, amplifying injury.

Although bilateral external ophthalmoplegia characterized anti-GQ1b antibody syndromes, ​​unilateral involvement represented a recognized variant phenotype​​, documented in approximately 27–31% of reported cases (3, 5). Notably, internal ophthalmoplegia (IO) is not uncommon. Lee et al. reported IO in 54.5% (6/11) of patients with acute ophthalmoplegia without ataxia, though all cases in their cohort presented bilaterally (3).​​ Our case and literature review identified IO in 6 of 18 cases (33.3%), with 4 cases exhibiting unilateral involvement. ​​However, the specific mechanisms driving unilateral manifestations remained incompletely understood.​​ Potential factors included heterogeneous ganglioside distribution or localized barrier disruption. Clinically, unilateral presentations may delay diagnosis, as isolated pupil-sparing third nerve palsy often raises concern for vascular or compressive lesions. Vigilance for antecedent infections and early serological testing remain crucial, as prompt recognition may prevent unnecessary invasive investigations while guiding targeted immunotherapy in severe or persistent cases.

Reviewing the available literature on this presentation, a tendency toward male predominance has been observed. Among the 18 patients with anti-GQ1b-associated unilateral ophthalmoplegia, 13 were male and 5 were female. This ​​observation was consistent with​​ findings from Choi et al., who reported that 7 out of 8 patients (88%) with anti-GQ1b antibody-positive single ocular motor nerve palsy were male. Furthermore, patients presenting with anti-GQ1b associated unilateral ophthalmoplegia appeared to be younger​​. The median age was 31 years in our review. Choi et al. alse noted that patients with single ocular motor nerve palsy and positive anti-GQ1b antibodies were significantly younger (42 ± 16 years) than their seronegative counterparts (58 ± 15 years, p < 0.05) (9). Pediatric cases further demonstrated distinctive features, exhibiting higher rates of unilateral ophthalmoplegia (pediatric 44.4% vs. adult 5.7%, p=0.01) and autonomic symptoms (27.3% vs. 2.8%, p=0.04) compared to adults (10).

MRI findings revealed nerve enhancement in only two cases (11.1%), both demonstrating specific cranial nerve involvement without broader abnormalities. This low incidence underscores that brain MRI is usually unrevealing in this syndrome but remains essential to exclude potential mimickers, such as brainstem stroke, Wernicke encephalopathy, and infectious, demyelinating, or autoimmune encephalitis (11).

Elevated CSF protein with normal cell count (albuminocytologic dissociation) is a valuable laboratory finding in anti-GQ1b antibody syndromes. However, ​​this finding was uncommon in acute unilateral ophthalmoparesis without ataxia, with only 14-18% AO patients exhibiting protein elevation (5, 11). In our literature review, CSF protein levels ranged from ​​23 to 97 mg/dL​​, with ​​mild elevation​​ observed in ​​7 out of 18 patients (38.9%)​​​. Crucially, normal CSF findings do not exclude the diagnosis of anti-GQ1b antibody syndrome, particularly in acute isolated ophthalmoplegia. Therefore, serological testing for anti-GQ1b IgG ​​might offer​​ greater reliability for early diagnosis compared to CSF analysis (5).

The therapeutic management and prognosis of unilateral ophthalmoplegia associated with anti-GQ1b IgG antibodies appeared​​ consistent with broader MFS patterns. ​​Treatment approaches remained empirical, ranging from conservative observation to immunomodulatory interventions such as corticosteroids and IVIg. ​​Notably, both treated and untreated patients indicated favorable outcomes in available reports​​. This consistented with San-Juan et al.’s analysis of 19 MFS patients (63% untreated), which ​​observed​​ no significant difference in recovery timelines between immunosuppression-treated and untreated groups, with symptom resolution typically occurring within 6 months (mean 35 days for ataxia, 93 days for ophthalmoplegia, 64 days for areflexia) (12). Similarly, Mori et al. ​​reported​​ complete recovery regardless of therapeutic intervention in all 50 MFS patients studied (13). ​​​​In our case,​​ the ​​initially inadequate response​​ to corticosteroids heightened patient and family anxiety, prompting the addition of IVIg therapy. IVIg ​​contributed to a modest clinical improvement and reduced recovery time, likely through mechanisms such as reducing anti-GQ1b antibody binding and mitigating their pathological effects (14). While these collective findings suggested that spontaneous resolution might characterize this phenotype, our experience underscored the critical importance of comprehensive management. Beyond immunotherapy, proactively addressing psychological distress is essential. This includes ​​detailed communication​​ regarding the expected protracted recovery timeline (often weeks to months), reassuranced about the generally favorable prognosis, ​​and realistic expectation-setting regarding treatment responses.​​ Such proactive communication is vital to mitigate anxiety, particularly when symptoms persist despite therapeutic efforts. Prospective studies with larger cohorts are warranted to​​ further delineate the natural history of this presentation and optimize strategies for psychological support.

This study has several limitations. ​​First,​​ the retrospective analysis is based solely on case reports and case series, which are inherently susceptible to publication bias and lack the methodological rigor of controlled studies. ​​Second,​​ for the majority of cases included, the timing of the lumbar puncture relative to symptom onset was not reported, limiting the interpretability of cerebrospinal fluid findings (e.g., albuminocytologic dissociation). ​​Finally,​​ some potentially relevant articles were excluded from our review due to insufficient or incomplete reporting of essential clinical details.

## Data Availability

The original contributions presented in the study are included in the article/supplementary material. Further inquiries can be directed to the corresponding authors.
